# Glances at the Hospitals

**Published:** 1904-01-09

**Authors:** 


### GLANCES AT THE HOSPITALS.

### ROYAL INFIRMARY, EDINBURGH.

The ordinary income amounted to £32,621, and the ordinary expenditure to £47,193, showing a deficiency of £14,572; but legacies and donations received during the year reached the respectable figure of £21,007. Of this sum £18,883 was passed on to the general fund, £1,000 to the extension fund, and £1,124 to the convalescent home. At the beginning of the year the infirmary contained 616 patients, and 9,345 were admitted during it, making a total of 9,961 under treatment. Of these 4,631 were discharged cured, 3,226 relieved, 595 for other reasons, 826 died, and 683 remained in the wards. The average number of nurses in residence was 199, and there were 656 applications for admission, showing that nursing is still a very popular profession. The infirmary is being considerably extended. One new pavilion is already finished, and the ear and throat and ophthalmological pavilions will be completed during the summer of 1903. The medical tables are exceedingly well arranged, and may be recommended as models to those infirmaries which have not yet adopted a satisfactory method of presenting statistics. Of acute rheumatism there were 98 cases, and of these 74 were cured, 19 relieved, and 3 died. The average duration of treatment was 40 days. The extraordinary number of 326 cases of acute pneumonia was under treatment, being an increase of 70 per cent. over the previous year. Of the number 193 were cured, 6 relieved, and 121 died, being at the rate of 37 per cent. There were 212 cases of phthisis. We do not know whether any special pavilions exist at this infirmary for the treatment of consumptive patients, but we greatly doubt the advisableness of such a number being housed with other infirmary cases. In the medical wards the percentage of deaths was 12·8, and the average stay in the hospital was nearly 28 days. In the surgical wards 5,800 patients were admitted, and 303 died, being a percentage mortality of 5·24. The total number of operations was 2,816, the deaths 189, and the percentage mortality 6·71. It is most unfortunate, and also incomprehensible, that while the medical tables are so well prepared, the surgical ones show no results. Even the table of operations tells us nothing beyond the number, age, and sex of the patients operated on for the various diseases. For instances there were 141 cases of laparotomy for various diseases, intussusception, excision of the appendix, etc., and to get at the results we have to refer to table C, which shows the deaths occurring after operation. Here we find that there were 16 deaths from appendicitis, and on referring back to the table of operations we learn that 75 cases were operated on. There is no anæsthetic table, and although chloroform is known to be the agent most often used north of the Tweed it would have been instructive to know whether death was attributed to its administration in any one of the thousands of cases in which it was used during the last year.

### TOTTENHAM HOSPITAL.

The receipts amounted to £6,461, being an increase of £2,000 on those of the previous year, and in addition legacies amounting to £325 were received. The expenditure was £5,034, so that the finances are in a satisfactory condition. The hospital draws its *clientèle* from a wide area embracing Tottenham, Edmonton, Ponder's End, Wood Green, Waltham Cross, Cheshunt, Southgate, Enfield, etc. It has land attached to it, and can well bear the contemplated enlargement for which money is now being collected. During the year there were 820 in-patients, and of these 669 were discharged, 110 died, and 41 remained under treatment. The daily average of beds occupied was 64. An important step has been taken by the foundation of a college for post graduate teaching in connection with the hospital. These lectures are open to all qualified medical men. The medical and surgical tables are unusually good. There were 31 cases of pneumonia, and of these 25 were cured and 6 died; 49 cases of rheumatic fever show 1 death. Ovariotomy was performed 9 times with 1 death. The total number of major and minor operations was 827. No anæsthetic table is given. The out-patients' department is very large, and 17,153 patients were seen, involving over 50,000 attendances.

### ABERDEEN ROYAL INFIRMARY.

The total ordinary income amounted to £8,454, and the total ordinary expenditure to £10,884, showing a deficiency of income of more than £2,400, and it would seem that something like an additional £3,000 a year is required to maintain the infirmary in its present efficient state. It is proposed to make a vigorous effort to induce the working people themselves to take more interest in the infirmary and support it better, and this is the right course to pursue. If these people do not subscribe better to an institution which exists chiefly on their account they can hardly expect other classes to make up their deficiencies. Lord Mount-Stephen has come forward in the most generous way and proposes to transfer securities to the infirmary which will provide another £1,000 a year, and he quite naturally and rightly expects the Aberdeenshire working men will provide the other £2,000 required. We trust it will be so. The average number of beds occupied was 211, and the cost per bed occupied was £49 6s. On January 1st the infirmary contained 202 patients, and 2,463 were admitted since. Of the total 1,045 were discharged recovered, 871 were discharged relieved, 212 died, and 202 remained in the wards. The average stay in the infirmary was 29 days. The medical and surgical tables are carefully compiled. There were collectively 12 cases of nephrectomy, nephrorrhaphy, and nephrolithotomy, and of these 9 were cured and 3 relieved. Of 19 cases of ovariotomy 15 were cured, 1 died, and 3 remained under treatment. One thousand four hundred and forty-seven general anæsthesias were conducted during the year, and there were 377 gas and ether anæsthesias. The number of operations performed on the in-patients was 1,054.

### NEWCASTLE-ON-TYNE SICK CHILDREN'S HOSPITAL.

THE income amounted to £3,662, which rather more than covered the expenditure for the year. An anonymous donor sent £1,000, and several smaller donations were received. A ball realised £160. The committee have been able to buy some additional land on which to build a much-needed extension of the out-patients' department, which is estimated to cost about £10,000, and towards which some legacies have already been received. The total number of in-patients was 801. Of these 442 were cured, 129 relieved, 62 died, 103 were not relieved, and 65 remained in the hospital. The average number resident was 62, and the average stay in the hospital was 31½ days. The average cost of each in-patient was £4 6s. 2d., which included all expenses. The medical and surgical tables are very well arranged, and the results can be picked out without difficulty. Of 12 cases of pneumonia 8 recovered, 3 died, and 1 remained under treatment. There were 27 cases of herniotomy resulting in 24 recoveries and three deaths.

